# Shoot organogenesis and somatic embryogenesis from leaf and petiole explants of endangered *Euryodendron excelsum*

**DOI:** 10.1038/s41598-022-24744-y

**Published:** 2022-11-28

**Authors:** Yuping Xiong, Shuangyan Chen, Teng Wu, Kunlin Wu, Yuan Li, Xinhua Zhang, Jaime A. Teixeira da Silva, Songjun Zeng, Guohua Ma

**Affiliations:** 1 Guangdong Provincial Key Laboratory of Applied Botany, South China Botanical Garden, The Chinese Academy of Sciences, Guangzhou, 510650 China; 2 Institute of Nanfan and Seed Industry, Guangdong Academy of Sciences, Guangdong, 510316 China; 3 Guangzhou Minghui Landscape Technology Development Co, Ltd., Guangzhou, China; 4 Independent Researcher, Ikenobe 3011-2, Miki-cho, Kagawa-ken 761-0799 Japan

**Keywords:** Biotechnology, Cell biology, Developmental biology, Physiology, Plant sciences

## Abstract

*Euryodendron excelsum* H.T. Chang is a rare and endangered woody plant endemic to China. It is very important to conserve and propagate this species from extinction. In this study, leaves and petioles from the axillary shoots in vitro were used as explants to culture on the different plant growth regulator (PGR) woody plant medium (WPM) and establish an efficient shoot proliferation and plant regeneration system. WPM supplemented with 1.0 mg/L 2,4-D induced callus dedifferentiated into buds and somatic embryos on various media,including PGR-free WPM. However, only adventitious shoots formed on WPM with 1.0 mg/L of cytokinins such as 6-benzyladenine (BA), kinetin (KIN) or thidiazuron (TDZ). When another cytokinin, zeatin, was used, somatic embryos were induced directly from From cut surface of these explants. Adventitious roots could be induced from both explants on WPM with 1.0 mg/L α-naphthaleneacetic acid (NAA). Somatic embryos cultured in PGR-free WPM or WPM with 0.2 mg/L NAA developed roots. Plantlets derived from somatic embryos were transferred to a peat: sand (1:1, *v/v*) substrate, and showed survival rates of 64.3% at 30 days and 54.6% at 90 days. Callus clumps with adventitious shoot buds that were transferred to WPM containing 1.0 mg/L BA and 0.2 mg/L NAA generated a mean 3.3 multiple shoots. Callus-derived shoots regenerated and rooted successfully (100%) on agar-free vermiculite-based WPM with 0.5 μM NAA after 30 d. Plantlets transplanted to peat soil: vermiculite (1:1, *v/v*) displayed the highest survival (96.7%) after three months.

## Introduction

*Euryodendron excelsum* H.T. Chang (Pentaphylacaceae) is endemic to China^1,2^. Previously, it was classified in the Ternstroemoideae subfamily (Theaceae)^3,4^. *E. excelsum* is only distributed in South China, and is native to Guangdong and Guangxi provinces^5,6^. It is mainly distributed in rural areas where it is negatively impacted by human activity^7,8^. Only two ancient *E. excelsum* trees are still alive in Yangchun city^8^. In recent years, local governments have attempted to preserve the species by using iron fences to prevent local villagers and animals from destroying living trees. Even though scientists recently found 235 plants in the wild, no seedlings were found^9^. As a result of its rarity, *E. excelsum* has been listed as a nationally first-class protected endangered plant and is also classified as an extremely rare species^10–12^.

It is difficult for *E. excelsum* populations to renew themselves naturally, and the growth of young seedlings is slow, especially in harsh environments, resulting in poor survival and weak ecological competitiveness^13^. There is thus a need to seek alternative forms of propagation and conservation to overcome the limitations of this plant’s sexual (seed) reproduction^9,14,15^, which is the main reproductive pathway in natural communities^16^. The seeds do not have a period of dormancy, so they need to be sown quickly, otherwise they rapidly lose vitality due to a rapid loss in seed moisture that erodes germination percentage to as little as 5%^17^. It has been established an axillary shoot proliferation and plant regeneration system for *E. excelsum*^18^; During its *ex vitro* rooting, there is wide fluctuation in the endogenous levels of indole acetic acid (IAA) and hydrogen peroxide (H_2_O_2_) during the formation of the root primordium, and transcriptomic analysis indicated that multiple differentially expressed genes are involved in adventitious root development and plant hormone signal transduction^19^.

In a follow-up to those studies, in this paper, we used leaves and petioles from in vitro shoots as explants, for the first time to successfully induce callus, adventitious buds and somatic embryos. The ability to establish a different regeneration protocol to that achieved in China would allow for a diversification of regeneration protocols for this endangered tree, which has multiple usage in furniture and construction industries. This protocol lays down a solid foundation for a regeneration system that can aid the environmental protection and sensible utilization of *E. excelsum* germplasm.

## Results

### Effect of PGRs on callus induction of adventitious shoot and somatic embyogenesis from leaf explants

**Figure 1:**
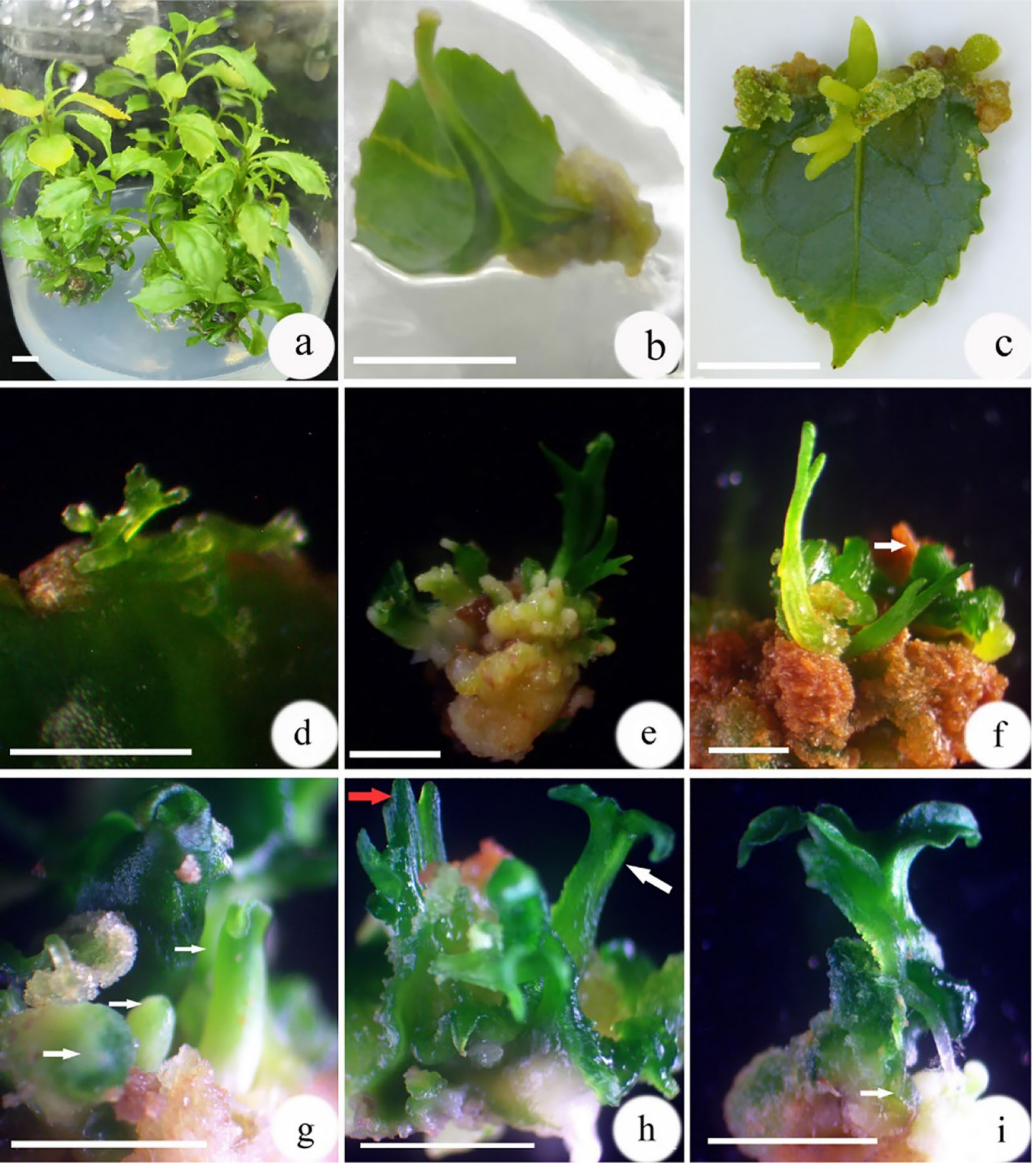
Shoot organogenesis from leaf explants in *Euryodendron excelsum*. (**a**) Leaf explants derived from axillary shoots on PGR-free WPM for 4 weeks. (**b**) Callus was induced on WPM with 1.0 mg/L 2,4-D after culture for 6 weeks. (**c**) Callus and adventitious roots were induced on WPM with 1.0 mg/L NAA after culture for 6 weeks. (**d**) Adventitious shoots were induced on WPM with 1.0 mg/L TDZ and 0.2 mg/L NAA after culture for 6 weeks. (**e**) Callus induced on WPM with 1.0 mg/L 2,4-D cultured for 6 weeks, then they were transferred to on WPM with 1.0 mg/L BA and 0.2 mg/L NAA for an additional 3 weeks, the callus differentiated into adventitious shoots. (**f**) Callus induced on WPM with 1.0 mg/L 2,4-D cultured for 6 weeks, then differentiated into adventitious shoots (white arrow) on PGR-free WPM after 21 d. (**g**) Callus induced on WPM with 1.0 mg/L 2,4-D cultured for 6 weeks, then differentiated into globular and torpedo-shaped somatic embryos (white arrows) on PGR-free WPM for 4 weeks. (**h**) Callus induced on WPM with 1.0 mg/L 2,4-D after culture for 6 weeks, then differentiated into adventitious shoots (red arrow) and somatic embryos (white arrows) on PGR-free ½WPM for 4 weeks. (**i**) Callus induced on WPM with 1.0 mg/L 2,4-D after culture for 6 weeks, then differentiated into cotyledon-shaped somatic embryo-like structure that developed roots on PGR-free WPM after 4 weeks. Bars = 1.0 cm.

When 1.0 mg/L 2,4-D was used, it induced light yellow and granular callus within 6 weeks, but callus was unable to directly differentiate (Fig. 1b). However, as the 2,4-D-induced callus was transferred to WPM medium containing others PGRs, such as 1.0 mg/L NAA, it turned yellow and became friable, while adventitious roots formed on leaf explants (Fig. 1c). On WPM supplemented with 1.0 mg/L TDZ, BA, or KIN and 0.2 mg/L NAA, very little callus was induced and it was compact and granular, but after 6 weeks, some adventitious shoots were visible among the callus (Table 1; Fig. 1d, e). When callus was transferred to the PGR-free WPM, both adventitious shoots and somatic embryos were visible on the surface of the callus (Fig. 1f). WPM supplemented with 1.0 mg/L zeatin induced more callus and more adventitious shoot buds than BA or TDZ. That callus was pale yellowish and granular, and after 6 weeks, a globular somatic embryo emerged from it (Fig. 1g–i). Among all cytokinins, most somatic embryos were induced by zeatin (Tables 1, 2). Generally, leaf explants formed more adventitious shoots and/or somatic embryos than petiole explants (Table 1).

**Table 1:** Effects of different PGRs on callus induction (after 6 weeks) and morphogenesis (after two months) from leaf and petiole explants of *Euryodendron excelsum*.

PGRs in WPM (mg/L)	Leaves		Petioles	
	Callus induced	Morphogenesis	Callus induced	Morphogenesis
2,4-D 1.0	+++++	Callus	++++	Callus
NAA 1.0	+++++	Callus with roots	+++	Callus with roots
KIN 1.0	+	Callus with shoots	+	Callus with shoots
TDZ 1.0	++	Callus with shoots	++	Callus with shoots
BA 1.0	++	Callus with shoots	+	Callus with shoots
Zeatin 1.0	+++	Callus with somatic embryos	++	Callus with somatic embryos

*Shoot proliferation and subculture on WPM supplemented with 1.0 mg/L BA and 0.2 mg/L NAA once every two months. Each treatment had 30 explants. For the treatment WPM with 1.0 mg/L 2,4-D, the number of explants was 180. + indicates a crude estimate of a relative amount of callus, ranging from sparse (+) to profuse (+++++).

**Table 2:** Differentiation into shoots and somatic embryos of callus induced by 1.0 mg/L 2,4-D for 6 weeks and then transferred to the different media for differentiation in *Euryodendron excelsum*.

PGRs in WPM (mg/L)	Morphogenesis	Number of shoots/callus clump	Number of somatic embryos/callus clump
PGR-free	Shoots and somatic embryos	1.8 ± 0.1 b	2.5 ± 0.2 c
NAA 1.0	Somatic embryos	0 c	3.7 ± 0.2 b
Zeatin 1.0	Somatic embryos	0 c	6.4 ± 0.4 a
TDZ 1.0	Adventitious shoots	3.4 ± 0.7 a	0 d
BA 1.0	Adventitious shoots	3.3 ± 0.7 a	0 d
KIN 1.0	Adventitious shoots	3.1 ± 0.6 a	0 d

*Each treatment had 30 callus clumps. Values represent means ± SD. Different letters within a column indicate significant differences according to Duncan’s multiple range test (*P* ≤ 0.05).

### Effect of PGRs on shoot organogenesis and somatic embryogenesis from petiole explants

**Figure 2:**
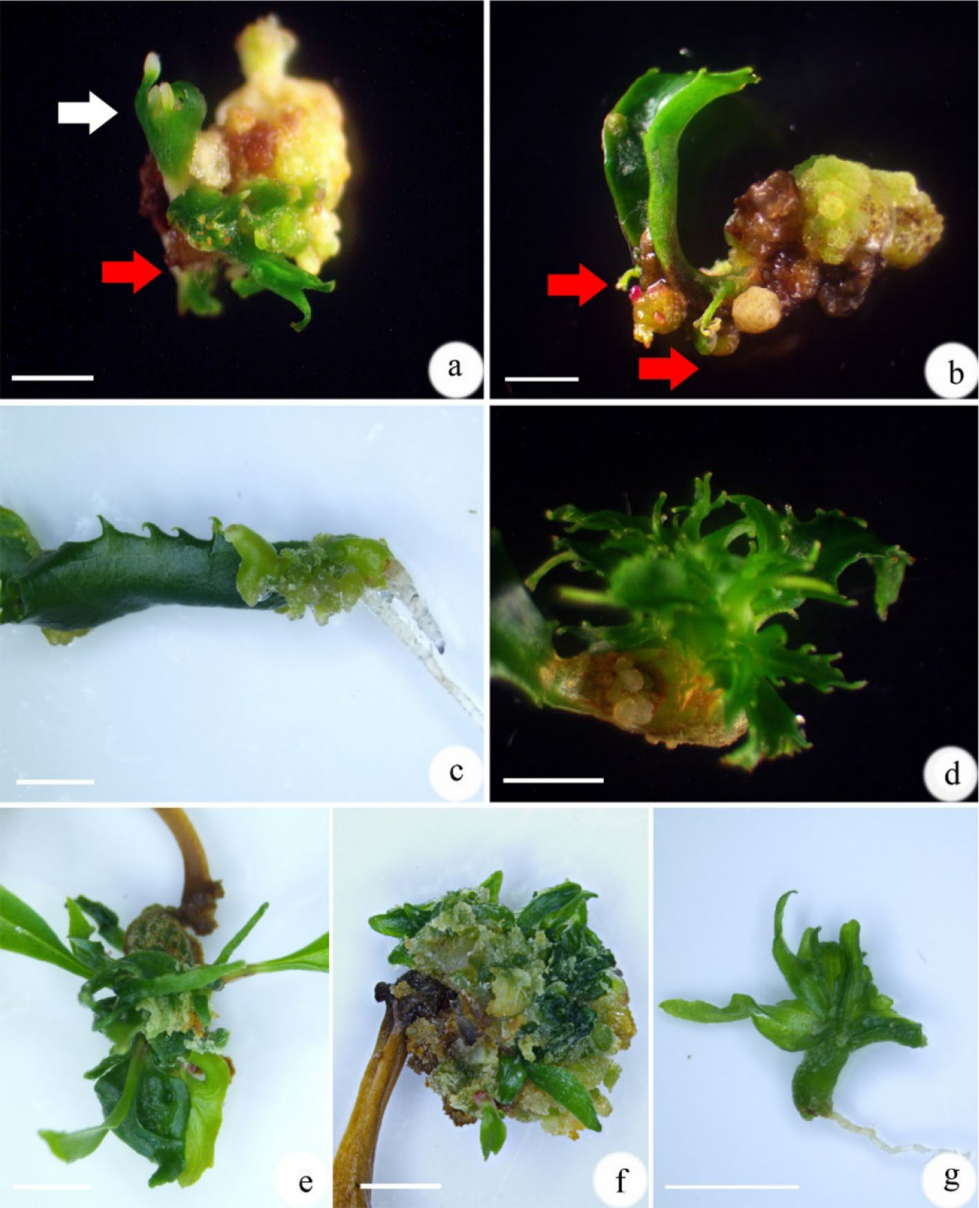
Shoot organogenesis from petiole explants of *Euryodendron excelsum*. (**a**) Callus induced on WPM with 1.0 mg/L 2,4-D after culture for 6 weeks, then differentiated into adventitious shoots (red arrow) and somatic embryo-like structure (white arrow) on PGR-free WPM for 3 weeks. (**b**) Adventitious shoots were induced on WPM with 1.0 mg/L TDZ after culture for 6 weeks. (**c**) Adventitious roots induced on WPM with 1.0 mg/L NAA after culture for 6 weeks. (**d**) Adventitious shoots induced on WPM with 1.0 mg/L BA after culture for 6 weeks. (**e**) A few adventitious shoots were induced on WPM with 1.0 mg/L KIN after culture for 6 weeks. (**f**) Fragile callus and somatic embryos induced on WPM with 1.0 mg/L zeatin after culture for 6 weeks. (**g**) A somatic embryos that was isolated from a callus clump derived from a petiole cultured on WPM with 1.0 mg/L zeatin for 6 weeks was transferred to WPM with 0.2 mg/L NAA for 3 weeks. Bars = 1.0 cm.

When Adventitious shoots were differentiated from the callus that was induced on WPM with 1.0 mg/L 2,4-D upon its transfer to WPM with 1.0 mg/L BA and 0.2 mg/L NAA (Fig. 2a). WPM supplemented with 1.0 mg/L NAA induced some adventitious roots and callus, but no adventitious shoots or somatic embryos were visible (Fig. 2b). WPM with 1.0 mg/L TDZ also induced some callus and adventitious shoots (Fig. 2c). WPM with 1.0 mg/L BA or KIN induce little callus and a few adventitious shoots (Fig. 2d, e). WPM supplemented with 1.0 mg/L zeatin also induced some callus that differentiated into somatic embryos (Fig. 2f). When culture period was extended to 6 weeks, some somatic embryos were observed (Fig. 2g).

### Differentiation of somatic embryos

**Figure 3:**
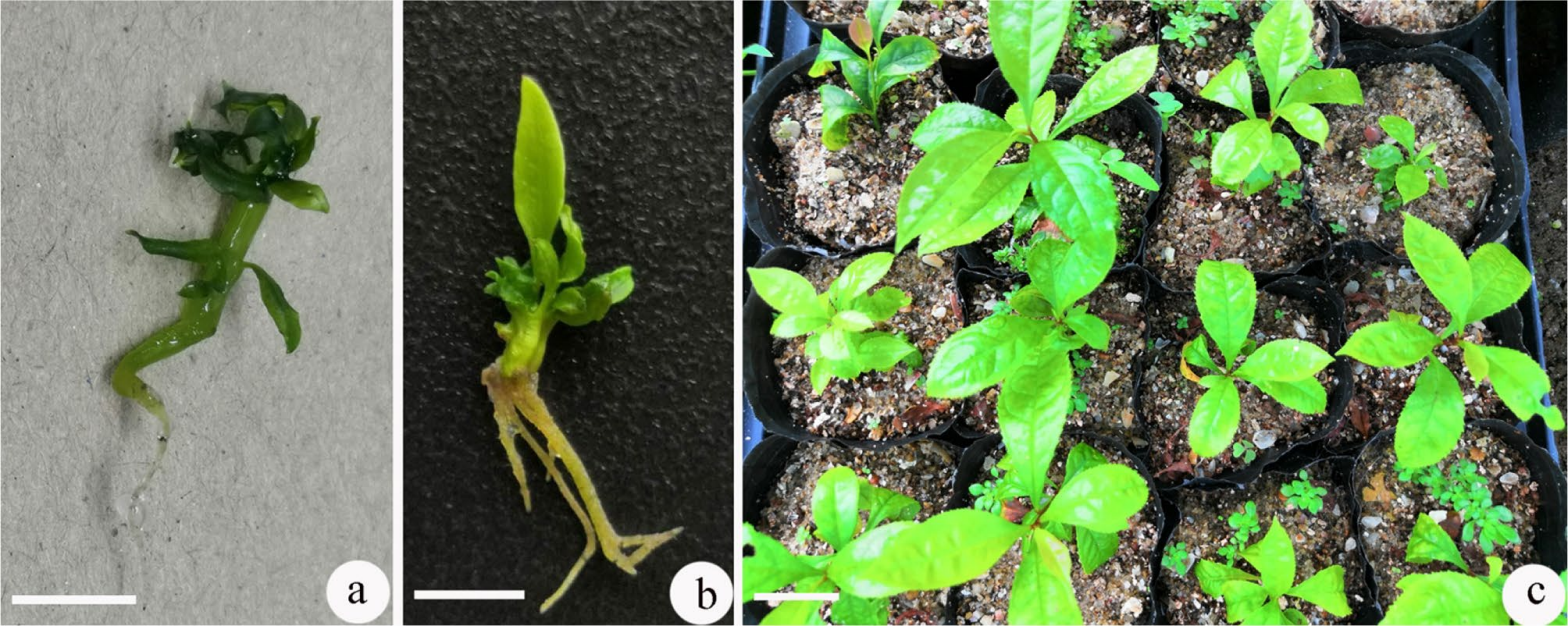
Recovery of *Euryodendron excelsum* somatic embryos and transplantation*.* (**a**) A single somatic embryo developed a radicle on WPM with 0.2 mg/L zeatin after 6 weeks. (**b**) Somatic embryos developed roots on WPM with 0.2 mg/L NAA after 6 weeks. (**c**) Somatic embryos from developed normal plantlets after three months. Bars: 1.0 cm (**a**, **b**); 5.0 cm (**c**).

When somatic embryos were induced on ½WPM with 1.0 mg/L 2,4-D, and were then transferred to PGR-free WPM for 3 weeks, some somatic embryos developed roots within 4 weeks (Fig. 3a). When somatic embryos were transferred to ½WPM with 0.2 mg/L NAA, roots developed at the base and some leaves formed at the apex of shoots within 6 weeks (Fig. 3b). When the above well-developed somatic embryos (total of more than 100 somatic embryos) (i.e., with well-formed roots and ample leaves) were transplanted to plastic bags with peat: sand (1:1, *v/v*), 64.3% survived within one month. Some developed leaves, but plantlets were comparatively small. After three months of growth only 54.6% of plantlets survived, but these plantlets developed normally (Table 3; Fig. 3c). This showed that regeneration via somatic embryogenesis was efficient.

**Table 3:** Transplantation of somatic embryos and rooting plantlets via axillary shoot of *Euryodendron excelsum*.

Tranplantation survival (%) in different regeneration pathways	Tranplantation period (months)	
	1	3
Somatic embryos	64.3 ± 1.2 b	54.6 ± 1.1 b
Rooted plantlets via axillary shoots	99.3 ± 0.7 a	96.7 ± 1.6 a

Trays were supplemented with peat: sand (1:1, *v/v*). Each treatment had 100 SELSs or plantlets. Values represent means ± SD. Different letters within a column indicate significant differences according to Duncan’s multiple range test (*P* ≤ 0.05).

### Acclimatization and transplantation

**Figure 4:**
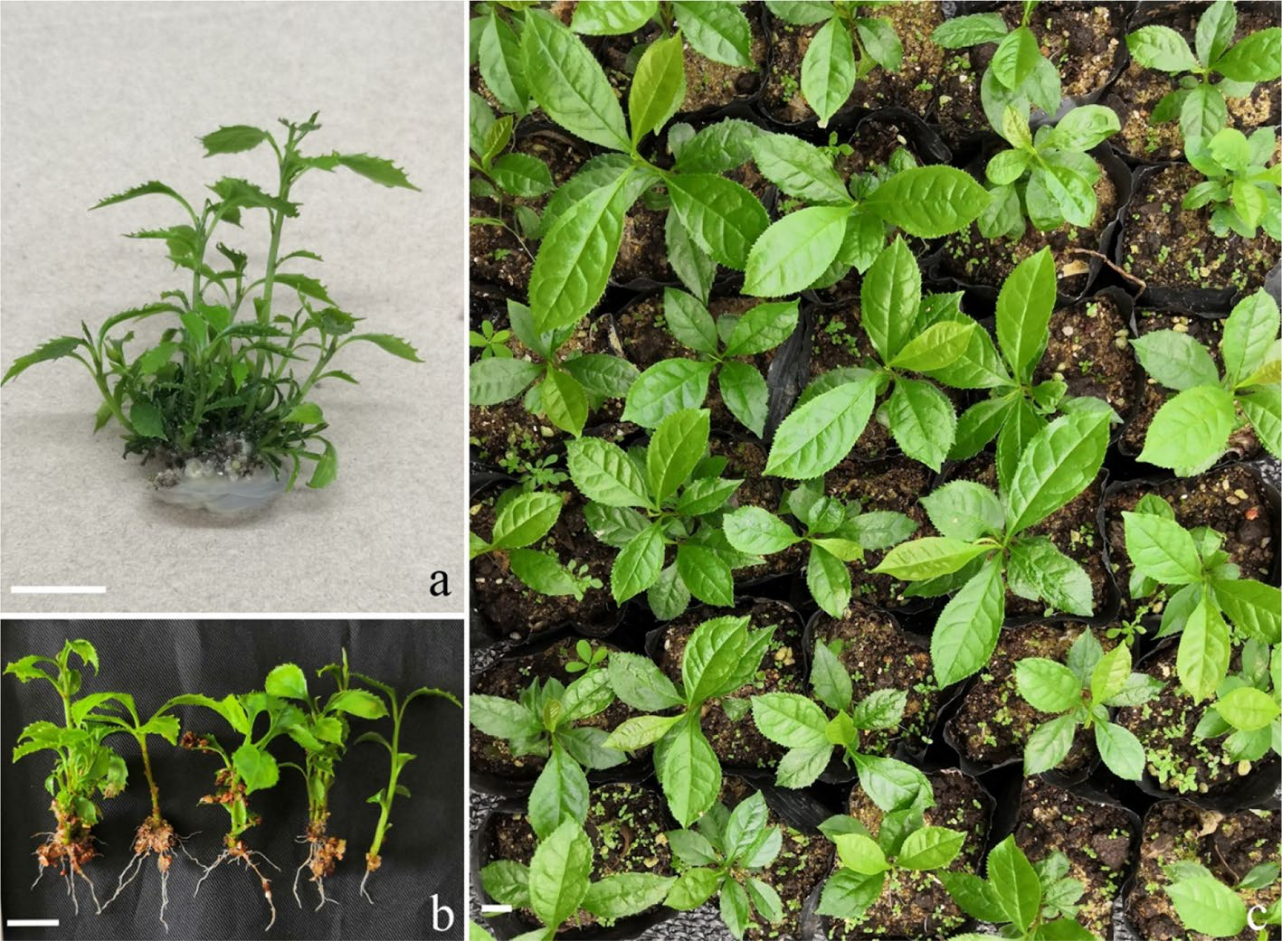
Adventitious shoot growth, rooting and transplanting in *Euryodendron excelsum.* (**a**) Callus clumps with adventitious shoots which was derived on WPM supplemented with 1.0 mg/L 2,4-D for 5 weeks then transferred to WPM supplemented with 1.0 mg/L BA for 5 weeks developed multiple shoots. (**b**) Shoots that were cultured on vermiculite-based WPM with 0.5 mg/L NAA for 2 months developed roots. (**c**) Plantlets were transferred to plastic bags with peat: sand (1:1, *v/v*) for three months. Bars = 1.0 cm.

Callus clumps with adventitious shoot buds that were induced on WPM with 1.0 mg/L 2,4-D were transferred to WPM with 1.0 mg/L BA and 0.2 mg/L NAA, inducing multiple adventitious shoots within 6 weeks (Fig. 4a). Individually separated shoots were transferred to vermiculite-based WPM with 5.0 μM NAA, and 100% of shoots developed roots within one month (Fig. 4b). Plantlets longer than 3 cm were transplanted to plastic bags with peat: sand (1:1, *v/v*), 99.3% of which survived after 1 month and 96.7% survived after 3 months (Table 3; Fig. 4c). This showed that survival percentage of rooted plantlets following transplantation was higher than that of somatic embryos- derived plantlets.

## Discussion

Morphogenesis from *E. excelsum* leaf and petiole explants need embryogenic callus induction and differentiation. Among several hormones, 2,4-D plays a major role in the induction and differentiation of embryogenic callus during plant regeneration in many woody plant species^20–24^. However, 2,4-D can inhibit callus dedifferentiation but when 2,4-D-induced callus is transferred to other media, the callus may dedifferentiate into organs.

In this study, when callus was transferred to PGR-free WPM, both adventitious shoots and somatic embryos formed: in WPM, in the presence of cytokinin zeatin or NAA, callus dedifferentiated into SELSs; in contrast, when callus was transferred to WPM with cytokinins BA or TDZ, only adventitious shoots were formed. This result is similar to *E. excelsum* (former family Theaceae species *Camellia nitidissima*), in which BA was best for the induction of adventitious shoots while zeatin most effectively induced somatic embryos^25^. Zeatin may induce the biosynthesis of nitric oxide in *Arabidopsis thaliana*^26^. In plants, TDZ can induce somatic embryogenesis^27–32^. TDZ has shown a dual-organogenic role in the induction of somatic embryos or in shoot organogenesis^33,34^, but may also result in developmental aberrations^35^. In *E. excelsum*, TDZ only induced adventitious shoot buds from leaves and petioles. Similar results were also seen in other woody plant species, such as *Acacia crassicarpa*^36^ and *Neolamarkia cadamba*^37^.

Expanding our previous study on axillary shoot proliferation and regeneration^18^, in this study, we induced SELSs for the first time. We also studied the recovery and transplantation of somatic embryos. Our results indicate more than 50% survival, suggesting a viable regeneration pathway via somatic embryos, but not as effective as rooting plantlets via axillary shoots. Possible reasons for the lower relative survival include: (1) Somatic embryos were relatively smaller and shorter (only 2 cm tall) than axillary shoots (generally 3 cm tall), which obviously improved plantlet transplantation survival percentage; (2) Somatic embryos usually need more time to develop true leaves (Fig. 3c), allowing the plantlets to grow in culture jars. An improved somatic embryo -based regeneration system needs to be optimized in the future.

Given that is a first-class endangered plant in China, the main objective in this communication was to establish a protocol that could allow for the mass propagation of genetic material, independent of the genetic stability of that material. This protocol allows for the mass propagation of tissue-cultured plants and the conservation of this rare and endangered genetic resource. Future analyses could employ ploidy analyses and use molecular markers to guarantee cytological and genetic stability, or not, but this depends on whether clonal material is needed (e.g. for landscaping) or genetically diverse material (e.g. for ecorestoration).

## Materials and methods

### Axillary shoot proliferation

The axillary shoots of *E. excelsum* were subcultured in South China Botanical Garden (Guangzhou, China) on Woody Plant Medium^38^ supplemented with 1.0 mg/L 6-benzyladenine (BA) and 0.2 mg/L α-naphthaleneacetic acid (NAA) for over four years^18^. Earlier seeds and seedlings were fetched and identified by Prof. Huagu Ye and planted in SCBG. He has collected available specimen (Number: 0276615–0276621) in SCBG herbarium. The axillary shoots originated from nodes culture from the stems of younglings which grew in South China Botanical Garden and it has been supported by our institute permission and all the collection comply with relevant institutional, national, and international guidelines and legislation and all methods performed in this study are in accordance with the relevant guidelines and regulations^18^.

As the stems developed, new axillary shoots were cut and transferred to fresh WPM with 1.0 mg/L BA, 0.2 mg/L NAA, 20 g/L sucrose and 6.0 g/L agar (Solarbio, Beijin, China) (pH 6.0) to proliferate shoots (Fig. 1a). All media were sterilized at 105 kPa and 121 °C for 20 min. Culture jars were placed in a 25 ± 1 °C culture room under a 12-h photoperiod with a photosynthetic photon flux density of 80 μM m^−2^ s^−1^ emitted by 40 W fluorescent lights (Philips, Tianjing, China)^18^. Each culture jar contained three multiple shoot clusters that were subcultured onto the same WPM every two months, and continuously subcultured on fresh WPM for over three years, allowing sufficient stock material to be produced (over 20-fold) in that period, for the following assays.

### Effect of plant growth regulators on induction of callus and shoots

Axillary shoots were proliferated on the WPM supplemented with 1.0 mg/L and 0.1 mg/L NAA for 6 weeks, then the multiple shoots were transferred to the PGR-free WPM for 4 weeks, to eliminate the effect of BA and NAA. Then the leaf and petiole explants from multiple shoots culture were inoculated onto several WPM-based media to induce callus and shoots (Table 1). After culture for 6 weeks, the induction of callus, shoots or SELSs was investigated. In the latter two, numbers were assessed per callus clump of the same size. Each treatment contained 10 jars with three leaf or petiole explants per jar. For WPM with 1.0 mg/L 2,4-D treatment, the number of leaf and petiole explants was increased to more than 180 for each treatment since the callus derived from this treatment was used for subsequent experiments. After culture for 6 weeks, the induction of callus, shoots or somatic embryos was investigated.

### Callus differentiation

Leaf- and petiole-derived callus originating from culture on WPM with 1.0 mg/L 2,4-D (Table 1) for 6 weeks was transferred to WPM-based media in an attempt to differentiate it (Table 2). Each treatment contained 10 jars with three callus clumps of the same size per jar. After 4 weeks, callus differentiation was investigated under a stereomicroscope (Nikon SMZ745T, Tokyo, Japan).

### Recovery of somatic embryos

Somatic embryos were cultured on PGR-free WPM with half-strength (micro- and macro-nutrients) (½WPM) or on ½WPM supplemented with 0.2 mg/L NAA or for 4 weeks to allow the somatic embryos to further differentiate and develop. Once somatic embryos were rooted and attained 2 cm in length, they were transferred to plastic bags (12 cm high; 10 cm diameter) with peat: sand (1:1, *v/v*) and placed in plastic tetragon trays (50 cm length; 30 cm width; 10 cm height) in the controlled greenhouse with the temperature at 20–28 °C, the maximum natural light intensity at 200 μM m^−2^ s^−1^ in the noon and relative humidity at 60–95%. The trays were watered with tap water once daily, every morning. One and three months after transplantation, the survival percentage was calculated as (number of plantlets that survived/total transplanted plantlets) × 100%.

### Rooting of adventitious shoots

Callus clumps from which adventitious shoots developed were transferred to WPM with 1.0 mg/L BA and 0.2 mg/L NAA for 6 weeks. Single shoots (3 cm high) were excised and inoculated onto WPM with 0.5 mg/L NAA and 11.0 g/L vermiculite per jar (5 shoots/culture jar).

### Acclimatization and transplantation

Plants that formed from adventitious shoots and that rooted well in vermiculite-based culture were transferred to plastic bags (12 cm high; 10 cm diameter) filled with peat: sand (1:1, *v/v*), with one plantlet per bag, and placed in a greenhouse under the conditions indicated above. Plastic bags were placed in plastic tetragon trays (50 cm length; 30 cm width; 10 cm height). Over 100 bags were sprayed daily with 100 mL of tap water at 8:00 a.m. One and three months after transplantation, the survival percentage was calculated as indicated above.

### Statistical analyses

Experimental data were statistically analyzed in SPSS 19.0 software (IBM, New York, NY, USA). After separating means—represented in tables as the mean ± standard errors (SE)—by analysis of variance, Duncan’s multiple range test (DMRT) was used to assess significant differences between means (*P* ≤ 0.05).

## Data Availability

Data is available upon reasonable request and Dr. Ma will response for request the data from this study. All data generated or analyzed during this study are included in this published article.

## Abbreviations

2,4-D: 2,4-Dichlorophenoxyacetic acid
BA: 6-Benzyladenine
KIN: Kinetin
NAA: α-Naphthaleneacetic acid
PGR: Plant growth regulator
TDZ: Thidiazuron
WPM: Woody Plant Medium (Lloyd and McCown 1980)
